# Effect of a Depolymerase Encoded by Phage168 on a Carbapenem-Resistant *Klebsiella pneumoniae* and Its Biofilm

**DOI:** 10.3390/pathogens12121396

**Published:** 2023-11-28

**Authors:** Xu Sun, Bingchun Pu, Jinhong Qin, Jun Xiang

**Affiliations:** 1 Department of Burn, Ruijin Hospital, Shanghai Jiao Tong University School of Medicine, Shanghai 200025, China; 15689072618@163.com; 2 Department of Immunology and Microbiology, Shanghai Jiao Tong University School of Medicine, Shanghai 200025, China; pbingchun@163.com (B.P.); jinhongqin@sjtu.edu.cn (J.Q.)

**Keywords:** depolymerase, phage, biofilm, carbapenem-resistant klebsiella pneumoniae

## Abstract

Infections caused by *carbapenem-resistant Klebsiella pneumoniae* (CRKP) are becoming increasingly common within clinical settings, requiring the development of alternative therapies. In this study, we isolated, characterized, and sequenced the genome of a CRKP phage, Phage168. The total genomic DNA of Phage168 was 40,222 bp in length, encoding 49 predicted proteins. Among these proteins, Dep40, the gene product of ORF40, is a putative tail fiber protein that exhibits depolymerase activity based on the result of bioinformatics analyses. In vitro, we confirmed that the molecular weight of the Phage168 depolymerase protein was about 110 kDa, the concentration of the produced phage 168 depolymerase protein was quantified as being 1.2 mg/mL, and the depolymerase activity was still detectable after the dilution of 1.2 µg/mL. This recombinant depolymerase exhibited enzyme activity during the depolymerization of the formed CRKP biofilms. We also found that depolymerase, when combined with polymyxin B, was able to enhance the bactericidal effect of polymyxin B on CRKP strains by disrupting their biofilm. When recombinant depolymerase was used in combination with human serum, it enhanced the sensitivity of the CRKP strain UA168 to human serum, and the synergistic bactericidal effect reached the strongest level when the ratio of depolymerase to human serum was 3:1. Our results indicated that depolymerase encoded by Phage168 may be a promising strategy for combating infections caused by drug-resistant CRKP formed within the biofilm.

## 1. Introduction

Bacterial drug resistance is a serious threat to global health, and effectively removing drug-resistant bacteria and breaking through their biofilms remain major challenges in clinical practice. According to the results of CHINET drug resistance surveillance in China [1], the detection rate of carbapenem-resistant *Klebsiella pneumoniae* (CRKP) has increased significantly in recent years. From 2005 to 2019, the detection rate of CRKP increased from 3% to 10.9% [2]. *Klebsiella pneumoniae* (KP) is an opportunistic pathogen that is widely distributed in the normal environment, colonizing the human oral cavity, skin, respiratory and digestive tracts [3]. Due to the widespread prevalence of CRKP in recent years, the Centers for Disease Control and Prevention (CDC) has recognized it as a major threat to public health [4]. The high morbidity and mortality rates of infectious diseases caused by CRKP have become a great challenge in terms of clinical prevention, control, and treatment. Therefore, CRKP and related research have become a topical issue in the field of anti-infectives.

Recent studies have shown [5] that several KP are adept at evading recognition by the host’s intrinsic immune system and are resistant to phagocytosis and killing by host cells due to a series of pathogenicity factors (including lipopolysaccharide (LPS), capsular polysaccharide (CPS), adhesins, and iron carriers, etc.), which are resistant to host cell phagocytosis. Among them, LPS and CPS, as important components of the outer membrane of KP, play an indispensable role in the relationship between KP and the host. CPS, a type of polysaccharide produced by KP, is considered to be an important virulence factor, which acquires higher virulence by preventing the phagocytosis of phagocytes. LPS is another pathogenicity factor that protects bacteria, also known as endotoxin, present in the bacterial outer membrane, which consists mainly of O-antigen, lipid A, and polysaccharides, and it has been shown [5,6,7] that LPS-deficient KP can be killed by complement-mediated immune effects. In addition to the virulence factors carried by KP itself, as described above, the mechanism of resistance caused by the biofilm it produces has also been a hot topic of research by scholars at home and abroad in recent years. A bacterial biofilm is a structure produced by a microbial community, which is a polysaccharide matrix adsorbed to the surface of a specific object by the bacterial community, reproduced, differentiated, and secreted, and the main components are bacteriophage (about 10%) and extracellular polymeric substance (EPS, about 90%). The EPS consists of polysaccharides, proteins, nucleic acids, and so on [8,9]. The production of a biofilm protects bacteria from fluid shear, assists bacteria in resisting harsh environments, and enables the bacterial community in the biofilm to attach more firmly to the surface of the implant. Compared to planktonic bacteria, bacteria in biofilms are significantly more resistant to antibiotics by about 10–1000 times [10]. In addition, biofilm-associated infections can inhibit the activation of host phagocytes and the complement system [11,12], which protects the invading bacteria from escaping the destruction of the immune system, eventually developing into chronic or refractory infections [13].

Currently, the systemic application of multiple sensitive antibiotics remains the only option for severely burned patients with drug-resistant KP infections, in addition to surgical treatment of wounds. However, the pathogenicity of KP capsules and the formation of biofilms make conventional antibiotics ineffective in controlling or eliminating the infection [14]. To overcome the severe drug resistance situation, researchers and clinicians have begun to explore traditional phage therapy. Generally, phage can rapidly release many daughter phages, as well as endotoxins, exotoxins, superantigens and other substances after acting on the target bacteria. These substances may cause serious adverse reactions to the human body, such as excessive inflammatory reactions and allergic reactions, so their long-term safety needs to be further understood. In contrast, phage-derived enzymes can specifically target highly conserved bacterial structures (polysaccharide components, etc.) in mammalian cells [15] and are generally considered safe in application. Among phage-encoded proteins, some phage tail fibrillar proteins (TFPs) have been found to contain specific depolymerizing enzymes, which have unique advantages, such as high stability and specificity, and they are involved in the inhibition of biofilm formation [16].

In our previous study, the CRKP UA168 strain (hereinafter referred to as UA168) was isolated from the deep venous catheter of a burn patient. UA168 was detected as an ST11-type strain in the clinical microbiology laboratory, and the clinical resistance results showed that UA168 was resistant to all antibiotics except polymyxin B. The biological characteristics of this strain have not been confirmed yet. In this study, we further discovered and characterized Phage168 as having the capacity to exhibit plaques that were surrounded by halo regions. Using Phage168, which has been fully genome sequenced, we cloned polysaccharide depolymerase in vitro through the use of molecular biology techniques and performed a series of experiments to study its coding function, enzymatic and depolymerase activities, as well as the effects of polymyxin alone or in combination with polymyxin and with or without the presence of human serum on CRKP strains and biofilms, providing early evidence of its function that will be potentially lead to a treatment for clinically multidrug-resistant KP.

## 2. Materials and Methods

### 2.1. Isolation and Purification of Phage

A sample of 15 mL of sewage from the sewage treatment center of Ruijin Hospital was filtered through a filter with a pore size of 0.22 µm and mixed with 100 µL of CRKP UA168 isolated from the blood of burn patients in Ruijin Hospital, and 8 mL of lysogeny broth (LB) culture solution at 3 times the concentration, and incubated overnight at 37 °C in a constant-temperature shaker. The overnight culture medium was centrifuged for 20 min, and the supernatant was extracted. The phage was isolated by the drop plate method [17], in which the lower plate was poured with an appropriate amount of solid LB medium, and the upper plate was poured with a mixture of 400 µL of host bacterial liquid and 3 mL of semi-solid LB medium at the appropriate temperature (around 45 °C). After centrifugation, 5 µL of the supernatant was dropped on the plate and placed in the incubator at 37 °C for 4 h. If transparent phage plaques could be seen, it was considered that the phage was successfully screened. The transparent phage spots were picked, and the phage was purified by the double-layer agar plate method [18]. A single phage spot was picked, and the above method was repeated three times to obtain the purified phage, which was named phage 168.

### 2.2. Transmission Electron Microscopy

An appropriate amount of Phage168 stored at 4 °C was centrifuged and dialyzed by cesium chloride density gradient [19], 100 µL of the dialyzed Phage168 was taken, and the supernatant was discarded after 40 min of centrifugation. The precipitate was washed with 10-fold diluted PBS buffer and fixed in a 2% glutaraldehyde solution for 30 min, centrifuged again as before, and the supernatant was discarded, and the precipitate was resuspended in diluted PBS buffer. The phosphotungstic acid negative staining method [20] was used to drop the immobilized Phage168 on a copper mesh, and the morphological structure was observed by 150,000 times transmission electron microscopy.

### 2.3. Genomic DNA Sequencing and Annotation

The DNA of Phage168 was extracted by DNeasy Blood & Tissue Kit (Qiagen, Nasdaq, NY, USA) and was subjected to high-throughput sequencing on the Illumina HiSeq 3000 platform (CHGC, Shanghai, China). The genome of Phage168 was annotated by the Rapid Annotation in Subsystem Technology (RAST; http://rast.nmpdr.org/ (accessed on 10 October 2022)). We searched for putative functional proteins that may encode polysaccharide depolymerase using online BLASTP on NCBI and focused on the tail fiber protein of the phage due to its highly homologous sequences with other phage depolymerase genes. Phage168 genome sequence and annotation information were uploaded to NCBI, and the GeneBank accession number was obtained.

### 2.4. Phylogenetic Analysis

To investigate the relationship between Phage168 and other phages with depolymerase, a phylogenetic tree was constructed using Molecular Evolutionary Genetic Analysis (MEGA). In view of the genomic mosaicism of phages, the phages were determined based on the amino acid sequences of the tail fiber protein, which were conserved among these phages. Additionally, the complete sequences of these phages used in the phylogenetic analysis were downloaded from the GenBank database based on BLASTp search results. ClustalW with default parameters and the neighbor-joining method with 1000 bootstrap replicates were used for multiple alignments of sequences and construction of the phylogenetic tree, respectively.

### 2.5. Cloning, Expression and Purification of the Recombinant Depolymerase

The phage tail fiber protein gene (open reading frame 40) was predicted to encode a putative polysaccharide depolymerase. The sequence of ORF40 was amplified from purified Phage168 by PCR using the specific primers F (5′-ATGGACCAAGATACTAAAAC-3′) and R (5′-TTAGGCGTTTAGGTAAACAC-3′) carrying restriction endonuclease sites Bam HI (New England Biolabs [NEB], Rowley, MA, USA) and Xho I (NEB), respectively. The amplified target product was cloned into the prokaryotic expression vector pET28a (Novagen, Madison, WI, USA) with a C-terminal His ×6 tag. The recombinant plasmid was verified by DNA sequencing, and then it was transformed into *E. coli* BL21 (DE3) competent cells. The plasmid-bearing cells were grown in 400 mL of LB supplemented with 50 µg/mL kanamycin at 37 °C with vigorous agitation to an optical density (OD600nm) of ~0.6. The recombinant protein was induced with 0.5 mM final concentration of isopropyl-β-D-thiogalactopyranoside (IPTG, Sangon Biotech, Shanghai, China), followed by shaking at 37 °C for 4 h. Next, the culture was harvested by centrifugation at 10,000 rpm and 4 °C for 10 min (Beckman, JA-10, Kraemer Boulevard Brea, CA, USA), and the pellet was resuspended in 20 mL of lysis buffer (20 mM Tris-HCl pH 7.9, 0.5 M NaCl, 10% Glycerol) containing 1 mM final concentration of phenylmethylsulfonyl fluoride (PMSF, Beyotime Biotech, Shanghai, China), a kind of complete protease inhibitor. The resuspended cells were lysed by a high-pressure homogenizer at 4 °C. The cell lysate was then centrifuged at 10,000 rpm for 10 min at 4 °C, and the supernatant was passed through a 0.22 um filter. The His-tagged protein purification was performed with a nickel-nitrilotriacetic acid (Ni-NTA) resin (Ni SepharoseTM 6 Fast Flow, GE Healthcare). After column equilibration with the washing buffer (20 mM Tris-HCl pH 7.9, 0.5 M NaCl, 10% Glycerol), the lysate was loaded and washed with 10 volumes of washing buffer. To determine the optimal elution buffer, the protein was eluted with elution buffer containing imidazole of different concentrations of 20 mM (NTA-20), 50 mM (NTA-50), 100 mM (NTA-100), 250 mM (NTA-250), respectively. Finally, the best-purified protein was eluted with 5 volumes of elution buffer (20mMTris-HCl pH 7.9, 0.5 M NaCl, 10% Glycerol, 250 mM Imidazole) according to the sodium dodecyl sulfate-polyacrylamide gel electrophoresis (SDS-PAGE) gels stained with Coomassie blue (Thermo Scientific, Waltham, MA, USA).

The protein eluted was dialyzed overnight at 4 °C in a bag of 10 kDa molecular-mass-cutoff membrane (Yobios, Xian, China) using 1000 volumes of dialysis buffer (20 mM Tris-HCl pH 7.9, 0.5 M NaCl, 10% Glycerol). The purified protein was concentrated by ultracentrifugation using a 10-kDa MW cut-off (MWCO) membrane (Millipore, Billerica, MA, USA). The BCA (Bicinchoninic Acid) method [21] was used to quantify the concentration of depolymerase. First, BCA working solution preparation was carried out by shaking and mixing liquid A and liquid B. The BCA working solution was configured according to the ratio of A:B = 50:1, and the gradient dilution of bovine serum albumin standard was performed, and the dilution solution was made with NTA-0, which was diluted into the standard samples with the final concentrations of 25, 125, 250, 500, 750, 1000, 1500, and 2000 μg/mL, respectively. A total of 0.1 mL of different concentrations of standard samples and protein samples to be tested were put into 96-well plates. Each well was added 200 µL of working solution, mixed and sealed thoroughly, placed at a constant temperature of 37 °C for half an hour, and then cooled down to room temperature, and the absorbance values of individual samples were measured by enzyme labeling instrument (A562) to draw the standard curve.

### 2.6. Depolymerization Activity Assay

200 µL of log-phase bacterial culture of UA168 was transferred onto 4 mL of LB soft agar overlay plate (LB with 0.7% agar) to form lawns. After drying, 10 µL of the purified serially diluted enzyme was spotted on the bacterial lawn, and the plate was incubated at 37 °C overnight. The formation of clear zones (halo) on the bacterial lawn was an indication of the antibacterial activity of the recombinant depolymerase. Additionally, depolymerase activity was assessed using the spot assay on UA168 and varying the enzyme concentrations from 120 µg/mL to 0.012 µg/mL. The elution buffer was used as a negative control.

### 2.7. Screening of Biofilm Producing Strains in CRKP

#### 2.7.1. Construction of Biofilm

After overnight cultivation in LB liquid medium, 12 CRKP strains (all from microbial cultures of blood from burn patients at Ruijin Hospital) were transferred to 5 mL LB liquid medium at a rate of 1:100 and sub-cultured once. The bacteria were shaken at 37 °C and 220 rpm until the absorbance OD600 value was around 0.4–0.6. The bacteria were diluted with sterile LB liquid medium to an OD600 value of 0.135–0.15. A total of 200 µL of diluted bacterial solution was taken from each well on a 96-well plate and allowed to stand at 37 °C for 48 h. Biofilm formation was observed in each well.

#### 2.7.2. Semi-Quantitative Detection of Biofilms

Crystal violet staining was used for semi-quantitative detection of biofilms [22]. When the biofilm reached the induction time, the culture solution in the 96-well plate was discarded, and each well was rinsed twice with 200 µL of PBS, followed by fixation with 100 µL of 10% methanol for 30 s per well. After aspirating the fixative, use 200 µL of 1% crystal violet per well and let it stand for 20 min (room temperature). Shake off the staining solution, wash each well with double-distilled water, and invert the Petri dish on absorbent paper until the water is absorbed. Decolorize each well by adding 0.1 mL of 33% acetic acid, mix well, and measure the absorbance (A570 nm). The blank control was repeated three times (equal volume of PBS).

#### 2.7.3. Effects of Depolymerase on Formed CRKP Biofilms

We found that CRKP strain UA168 was able to form a distinct biofilm (highest absorbance value). Strain UA168 was cultured overnight in LB liquid medium and then transferred 1:100 to 5 mL of LB liquid medium for one passaging, shaking the bacteria at 220 rpm at 37 °C until the absorbance OD600 value was 0.4–0.6, and then diluted with sterile LB liquid medium until the OD600 value was 0.135–0.15, and then 200 µL of diluted bacterial solution was taken from each well of a 96-well plate, and cultured in the incubator at 37 °C for 72 h. Biofilm formed in the 96-well plate was visible, and then the supernatant was removed and washed with 200 µL of PBS for 2–3 h. Then, PBS (positive control group) and 10 µg/mL depolymerase protein (depolymerase group) were added, respectively, and incubated at 37 °C for 2 h. The supernatant was removed. Crystalline violet staining [22] was applied to observe the effect of depolymerase on the formed CRKP biofilm. PBS solution was added to the unformed biofilm wells in this experiment as a negative control group.

#### 2.7.4. Antibiofilm Activity Combined with Antibiotics

To evaluate the activity of depolymerization enzyme in combination with antibiotics on UA168 biofilm, the experiment was divided into 4 groups with 3 replicate wells each. Group A was the control group. Each well was treated with 100 µL of PBS for 24 h. Group B was the depolymerase group. Each well was treated with 90 µL of PBS and 10 µL of depolymerase for 24 h. Group C was a combined group where each well was first treated with 10 µL of depolymerase, placed in a 37 °C thermostat for 2 h, and then treated with 10 µL of polymyxin B (256 µg/mL) and 80 µL of PBS solution for 22 h. Group D was a polymyxin B group, where each well was incubated for 24 h with the addition of 10 µL of polymyxin B (256 µg/mL) and 90 µL of PBS solution. Growth control and sterile control groups were also included. The effect of depolymerase and polymyxin B on CRKP biofilm was observed by crystalline violet staining after incubation at 37 °C for 24 h.

#### 2.7.5. Serum Killing Assay

To know if the depolymerase had a synergistic effect with serum to kill the bacteria and to determine the optimal volume ratio of serum to enzyme-treated bacteria in a killing assay, an experiment was conducted. The UA168 was cultured to log-phase, which was approached to 10^7^ CFU/mL. A total of 1 mL of the bacteria was centrifugated for 10 min at 8000 rpm, the pellet was washed with PBS for one time, and then the pellet centrifugated was resuspended by 500 µL PBS with or without 10 µg/mL enzyme. Then, the mixture was incubated at 37 °C for 1 h. It was then added to human serum from healthy donors at volume ratios of 3:1, 1:1, or 1:3 to a final reaction volume of 100 µL. To evaluate the role of serum complement in the serum killing assay, human serum was heated to 56 °C for 30 min to inactive the serum complement. The enzyme-treated and non-treated bacteria were added to inactive serum at a volume ratio of 1:1. All the samples were incubated for 3 h at 37 °C, and the bacteria were diluted in serial 10-fold to appropriate concentrations on LB agar plates. The effect of the combination of depolymerase and serum was expressed as the CFUs of bacterial reduction. The untreated bacteria and the PBS were incubated as a growth control and blank control. The serum was proved sterile. All the experiments were conducted independently twice, and the results were presented as means ± SD.

### 2.8. Statistical Analysis

Qualitative data were analyzed using a chi-square test and two-sided Fisher’s exact test. One-way analysis of variance (ANOVA) *p* values less than 0.05 were considered statistically significant for normally distributed data. All tests were performed using SPSS 26.0 (Chicago, IL, USA). All figures were created using GraphPad Prism 8.

## 3. Results

### 3.1. General Microbiological Characteristics of Phage168

**Figure 1:**
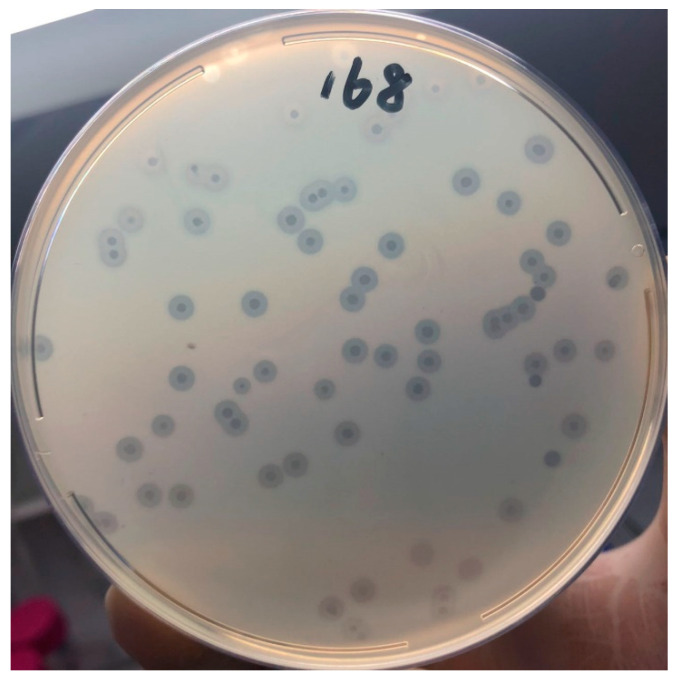
The phage plaques of Phage168. The phage plaques of Phage168 were morphologically homogeneous in size and surrounded by a translucent ring.

**Figure 2:**
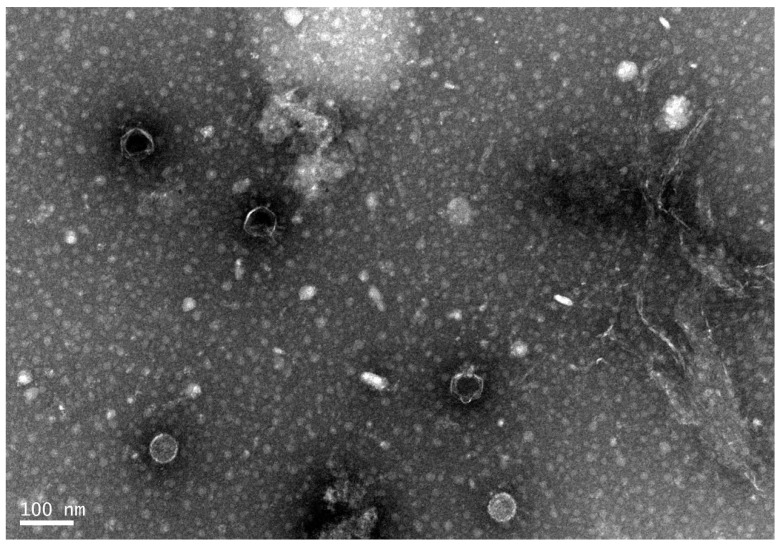
Phage168 observed by transmission electron microscopy. The head of the phage was dodecahedral, and the phage head was separated from the tail sheath by a collar. The tail was shorter. Based on these morphological features, the phage belongs to the family Podoviridae of Caudovirales. (scale bar: 100 nm).

The purified Phage168 showed translucent phage spots on bilayer plates, which were round and relatively uniform in size, surrounded by a translucent ring, and the halo continued to expand over time, which suggests the activity of a polysaccharide depolymerase, as shown in Figure 1. The head of the phage was dodecahedral, and the phage head was separated from the tail sheath by a collar. The tail was shorter. Based on these morphological features, the phage belongs to the family Podoviridae of Caudovirales (International Committee for the Classification of Viruses) (Figure 2).

### 3.2. Analysis of the Genome of Phage168

**Figure 3:**
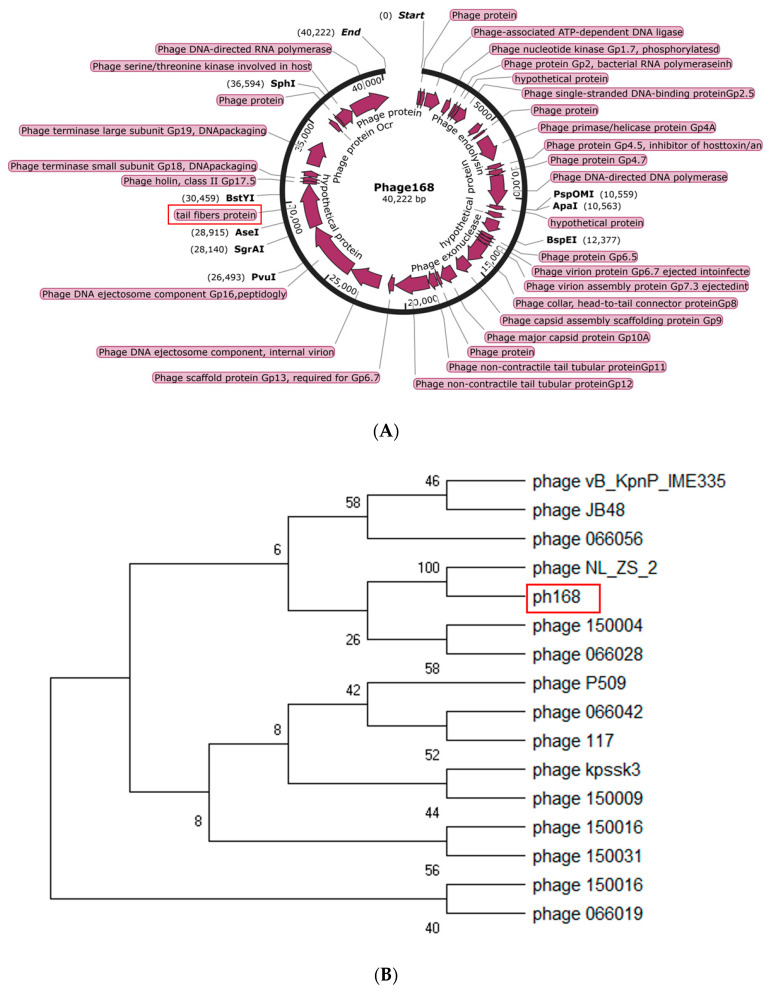
(**A**) Rast was used to annotate the entire genome of Phage168 bacteriophage (http://rast.nmpdr.org/ (accessed on 10 October 2022)). The genome of Phage168 was 40,222 bp in length, which encoded 49 predicted proteins. Among these proteins, Dep40, the gene product of ORF40, is a putative tail fiber protein, which is hypothesized to have depolymerase activity based on bioinformatics analyses. (**B**) Analysis of the phage 168 phylogenetic tree based on terminase large subunit sequences. Numbers next to branches are bootstrap values and represent confidence levels (%).

Whole genome sequencing analysis demonstrated that Phage168 has a linear double-stranded genome of 40,222 bp, including 49 predicted proteins (Figure 3A). We translated the DNA sequence of ph168 tail fiber with depolymerase into a protein sequence and blasted it on NCBI by BLASTp; homologous tail fiber protein sequences of other phages were identified from the GenBank database. Then, we chose 15 phages randomly and gained their complete genome for the construction of a phylogenetic tree. As shown in Figure 3B, ph168 and phage NL_ZS_2 were on the same branch, which meant that ph168 was closely related to phage NL_ZS_2, belonging to the family Podoviridae of Caudovirales order, revealing the homology between ph168 and NL_ZS_2. Further referring to the article about phage NL_ZS_2 [23], the phage was a kind of lytic phage against the multidrug-resistant *K. pneumoniae* isolates, especially ST11 Kp strains. We suspected that phage ph168 may have the same function on lysing ST11 antibiotic-resistant Kp strains. The GeneBank accession number for phage 168 is OR684907.

### 3.3. Depolymerization Activity Assay

**Figure 4:**
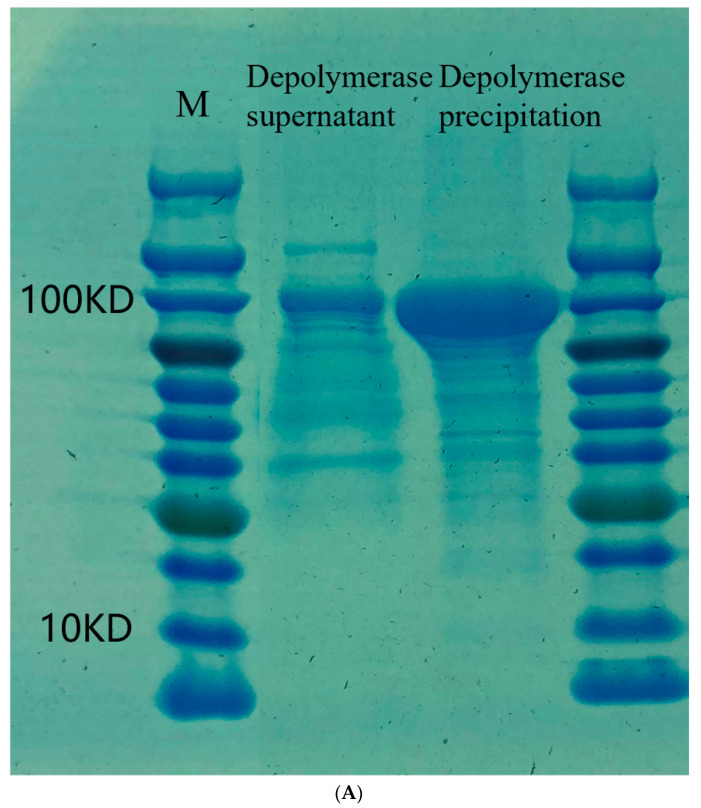

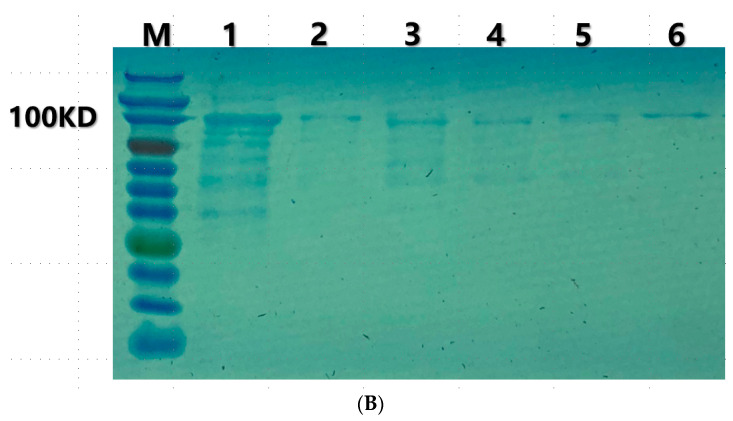
(**A**) SDS-PAGE electrophoresis of the induced depolymerase protein. (Note: M: protein marker; lane 1: depolymerase supernatant (after IPTG induction and expression); lane 2: depolymerase precipitation (after IPTG induction and expression)). (**B**) SDS-PAGE electrophoresis of purified protein eluate (Note: M: protein marker; 1: stock solution before purification; 2: NTA-0 eluate; 3: NTA-20 eluate; 4: NTA-50 eluate; 5: NTA-100 eluate; 6: NTA-250 eluent).

**Figure 5:**
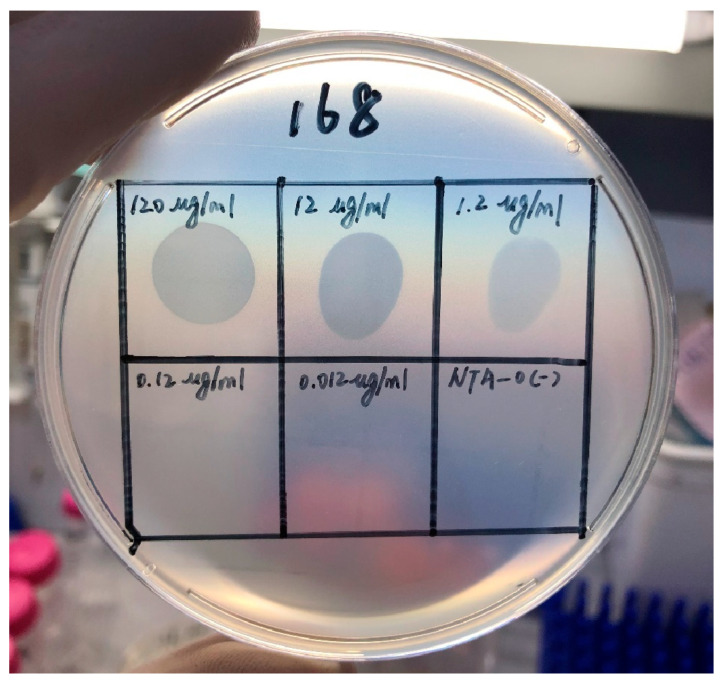
Protein spotting experiments with different concentrations of depolymerase. The presence of transparent halos can also be observed when the protein concentration reaches as low as 1.20 µg/mL. From left to right, 120 µg/mL, 12 µg/mL, 1.2 µg/mL, 0.12 µg/mL, and 0.012 µg/mL point to the double-layer agar plate with host UA168, NTA-0 as the negative control.

Depolymerase was clonally expressed, and SDS-PAGE in 12.5% gel showed the protein as 110 kDa (Figure 4A). Proteins were purified, and SDS-PAGE electrophoresis demonstrated a reduction in impurity protein bands (Figure 4B). Based on the relationship between the standard protein concentration and the absorbance value of A562, the formula for calculating the protein concentration was derived as Y = 0.0008261xX + 0.03920, and the OD value of the purified protein was 1.209. According to the standard curve, the concentration was 1.20 mg/mL. As shown in Figure 5, the presence of a transparent halo was also observed when the concentration of the depolymerase was 1.20 µg/mL, which indicated that the depolymerase concentration was still active when diluted to 1.20 µg/mL.

### 3.4. Depolymerase Could Destroy the CRKP-Formed Biofilm

**Figure 6:**
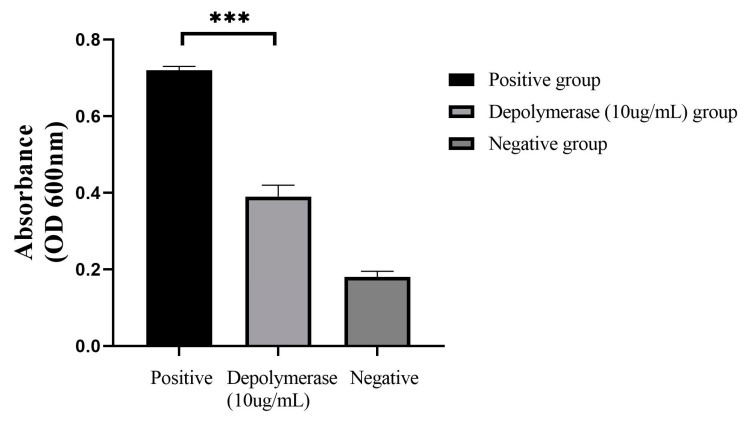
The effect of depolymerase on biofilm of CRKP strain (UA168). Compared with the positive group, the absorbance value of the depolymerase group decreased (0.33 ± 0.02), with a significant difference (*p* < 0.01) (Positive group: CRKP biofilm + PBS; Depolymerase group: CRKP biofilm + Depolymerase; Negative group: PBS; *** *p* < 0.001).

The biofilm of strain UA168 was successfully constructed, and the formed biofilm was treated with depolymerase protein (depolymerase group) and PBS (positive group), respectively. The results are shown in Figure 6. After 72 h of CRKP biofilm was incubated and treated with 10 µg/mL depolymerase protein, compared with the positive group, the absorbance value of the depolymerase protein group was reduced by (0.33 ± 0.02), with a significant difference (*p* < 0.01) The results indicated that the depolymerase could destroy the formed CRKP biofilm to a certain extent.

### 3.5. The Effect of Depolymerase Can Be Combined with That of Polymyxin B on CRKP Biofilm

**Figure 7:**
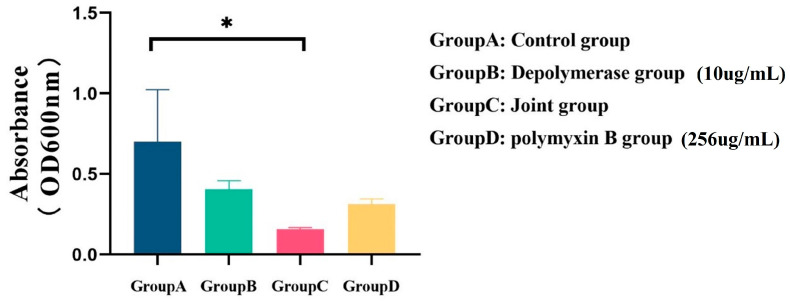
The effect of depolymerase and polymyxin combined application on biofilm (UA168). Compared with Group A, the absorbance of Group D decreased (0.39 ± 0.13); The absorbance of Group B decreased (0.29 ± 0.12). When 256 µg/mL polymyxin B was combined with a final concentration of 10 µg/mL depolymerase, its absorbance value decreased (0.54 ± 0.13), and Group C showed a significant difference compared to Group A (*p* < 0.05), although the absorbance of the Group B and Group D decreased compared to the Group A, there was no statistical difference, and the absorbance of the depolymerase group was higher than that of the polymyxin B group (0.09 ± 0.14). (* *p* < 0.05).

The combination of polymyxin B and depolymerase was further tested to determine whether there was a synergistic effect on the anti-CRKP biofilm. Here, our results (Figure 7) showed that compared with Group A, the absorbance of Group D decreased (0.39 ± 0.13); The absorbance of Group B decreased (0.29 ± 0.12); When 256 µg/mL polymyxin B was combined with a final concentration of 10 µg/mL depolymerase, its absorbance value decreased (0.54 ± 0.13), and the group C showed a significant difference compared to the Group A (*p* < 0.05); However, although the absorbance of the Group B and Group D decreased compared to the PBS group, there was no statistical difference, and the absorbance of the depolymerizing enzyme group was higher than that of the polymyxin B group (0.09 ± 0.14). Altogether, the above results indicated that when combined with polymyxin B, depolymerase proteins could enhance the bactericidal effect of polymyxin against CRKP strains by disrupting the biofilm.

### 3.6. Depolymerase Can Enhance the Serum-Mediated Bactericidal Effect on CRKP

**Figure 8:**
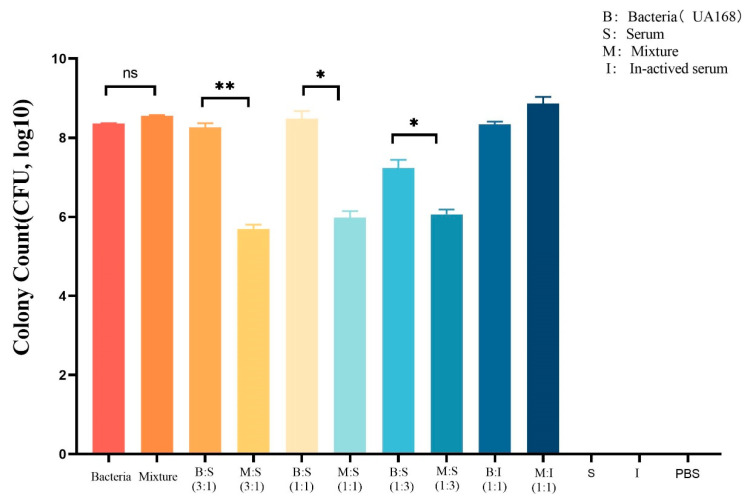
The synergistic bactericidal effect of depolymerase protein combined with human serum on strain UA168. Depolymerase enhanced the sensitivity of CRKP strain UA168 to human serum, and when the ratio of depolymerase to human serum was 3:1, the bacterial colonies were reduced the most, and the synergistic bactericidal effect was the strongest. When serum was inactivated, there was no significant change in the number of bacteria with or without depolymerase. The serum group, inactivated serum, and PBS groups showed no bacteria, and the experimental results were credible (ns: No significant difference; * *p* < 0.05, ** *p* < 0.01).

As shown in Figure 8, compared with UA168, which was not treated with depolymerase (hereinafter referred to as the bacteria group), the number of UA168 was significantly reduced when treated with depolymerase and different volumes of serum. When the bacterial: serum ratio was 3:1, the number of bacterial colonies was (8.26 ± 0.15) and (5.70 ± 0.15) log10 CFU, respectively. When the serum content increased to 50% (bacteria: serum is 1:1), the bacterial colony count decreased from (8.48 ± 0.28) log10 CFU to (5.98 ± 0.24) log10 CFU after treatment. When the serum content increased to 75% (bacterial: serum is 1:3), the bacterial colony count decreased from (7.23 ± 0.29) log10 CFU to (6.07 ± 0.16) log10 CFU after treatment; all decreases in the above three mixtures were statistically significant (*p* < 0.05). Additionally, compared among the bacteria group (8.36 ± 0.14) log10 CFU) and the host bacteria and depolymerase mixed group (8.55 ± 0.33) log10 CFU), the bacterial load in all groups with serum added was significantly reduced (*p* < 0.05). In addition, as the proportion of depolymerase protein increases, the number of colony reductions also increases. When the depolymerase content was at 75%, 50%, or 25%, the group treated with depolymerase had a colony decrease of (2.56 ± 0.15), (2.51 ± 0.26), and (1.17 ± 0.24) log10 CFU compared to the retrospective untreated control. However, when the inactivated serum acted on the host bacteria alone or together with the depolymerizing enzyme, the number of bacteria almost seemed to have neither decreased nor slightly increased, indicating that the incorporation of the serum played an important role in this experiment, which were shown in the last two groups in the figure. Furthermore, the serum, the inactivated serum, and the PBS were proven sterile, explicating that the test was credible. Thus, we confirmed in the vitro study that the depolymerase protein enhanced the sensitivity of CRKP strain UA168 to human serum, and the synergistic bactericidal effect reached its strongest level when the ratio of depolymerase to human serum was 3:1.

## 4. Discussion

Many studies have shown that polysaccharides on the bacterial surface, including CPS and EPS, are crucial to bacterial resistance, biofilm formation, and interaction with bacteriophage depolymerase [16]. Traditional antibiotics are not effective enough to control or eliminate infections due to multi-drug-resistant strains and bacterial biofilm-mediated stubborn infections [24,25,26,27]. Researchers have gradually turned their attention to bacteriophages and their derivatives [28,29,30,31]. Depolymerase can reduce bacterial virulence by destroying the polysaccharide capsule and biofilm of negative bacteria, which has become a hot spot in the field of infection research [32,33,34].

It has been found that the presence of a translucent halo around the plaque usually indicates that the phage possesses a depolymerizing enzyme, and the halo area can continue to expand over time as the plaque size remains constant [35]. In this study, we screened PHAGE168 using a previous collection of a CRKP and found that a transparent halo could be formed around PHAGE168, suggesting its potential depolymerization activity. Through bioinformatics prediction, we further found that phage 168 was highly homologous to phage NL_ZS_2, which was previously reported to have a killing effect on drug-resistant Klebsiella pneumonia, and we conjectured that Phage168 also had the same functional gene. The phage tail fiber protein gene sequence (ORF40) was put into the CDD module of NCBI, and after comparison, two conserved structural domains were found. One is the tail spike TSP1/Gp66 receptor binding N-terminal domain (E value: 8.60 × 10^−6^), and the other is the phage tail fiber protein (E value: 3.73 × 10^−7^). These two domains were found to have the activity of adsorbing to the host and releasing depolymerase in previous studies [36,37], so we also assumed that ORF40 is a gene with the function of depolymerase.

To further determine the activity of depolymerase, we first cloned the tail fiber gene fragment into the prokaryotic expression vector pET28a in our study and successfully expressed the polymerase protein after constructing the recombinant plasmid. We found in SDS-PAGE protein electrophoresis that the depolymerase protein was present in the supernatant and precipitate of the induced expression solution. Since the possible presence of inclusion bodies in the precipitate was not conducive to the next step of purification, the supernatant was selected for nickel column purification and ultrafiltration. 

Studies have shown that bacterial exopolysaccharides can be involved in the inhibitory effect of biofilms on the natural immune response [38], and polysaccharides are attached to the bacterial surface to form a complex network of scaffolding structures under electron microscopy. The scaffolding structure of the biofilm matrix remains after the use of antibiotics [39] and may harbor dormant bacteria [40,41]. In this case, antibiotics can only reduce the bacterial load. Phage depolymerase can act on the glycosidic bonds of EPS in bacterial biofilms to break down it [42]. In view of this hypothesis, extensive studies have been carried out by scholars. Gutiérrez et al. [43] suggested that the depolymerase Dpo7 could inhibit the formation of staphylococcal biofilm. Treatment of staphylococci with Dpo7 depolymerase from phage vB_SepiS-phiIPLA7 resulted in more than 90% removal of biofilm-adherent bacteria when Dpo7 was 0.15 u/mol, as measured by OD value. Similarly, Wu et al. isolated and purified dep42 from phage SH-KP152226 and used it on 226 KP strains. Compared with the control, 10 µg/mL Dep42 treatment of KP significantly reduced colonization in the biofilm but could not eliminate the biofilm [44]. In our study, the treatment of mature CRKP biofilms with 10 µg/mL depolymerase protein resulted in a decrease in absorbance values in the depolymerase group (0.33 ± 0.02) compared to the control group (PBS group). This indicated that depolymerase significantly reduced colonization in the biofilm and failed to eliminate the biofilm, which is also consistent with findings previously reported in the literature. However, Crystalline violet staining simply reflects the total biomass of the biofilm, and the disruption of the biofilm components with depolymerase results in a decrease in the stability of the biofilm, which may lead to the release of bacteria from the biofilm, or it may simply lead to a decrease in the biofilm components without altering the bacterial population. Therefore, the exact amount of bacterial load reduction should be further explored using plate counting for bacterial enumeration in future research.

Previous studies have also reported that depolymerase combined with antibiotic or immune serum therapy can further eliminate or disperse bacteria in biofilms, so to explore the role of Phage168 depolymerase in combination application, we first chose to combine the antibiotic polymyxin B, which is relatively sensitive to UA168. It has been previously reported in the literature that the depolymerase of phage KPO1K2 inhibited the biofilm formation of Klebsiella pneumoniae B5055 in combination with gentamicin [45]. The purified *P. aeruginosa* phage depolymerase PA3, in combination, decreased the minimum inhibitory concentration (MIC) and Minimum Bactericidal Concentration (MBC) of *P. aeruginosa* against four specific sensitive antibiotics to different degrees. Our study demonstrated that when depolymerase and polymyxin B were used together, depolymerase disrupted the CRKP biofilm, and this facilitated the entry of polymyxin B to exert its bactericidal effect, and the two did have a synergistic effect, which is also consistent with previous reports [46]. However, the absorbance of the depolymerase group and polymyxin B group decreased but was not statistically different from that of the PBS group. The reason may be that the crystal violet staining in the semi-quantitative method of biofilm indirectly reflects the total amount of biofilm, including bacterial fragments, nucleic acids, polysaccharides, and other components, and the application of depolymerase led to the destruction of the polysaccharide components of the biofilm, the structure of the biofilm was altered or cracked, and the bacteria were released from the biofilm, which all these factors may make the absorbance appear high, so further plate counting of the number of live bacteria in the biofilm is needed to verify the results. In addition, polymyxin B with a concentration of 256 µg/mL was applied in our experiment, and whether other concentrations of polymyxin B combined with depolymerase have significant effects needs to be further explored.

CPS is an important virulence factor of Klebsiella pneumoniae capsular, which protects the host immune response through several mechanisms, including inhibition of phagocytosis by immune cells and inhibition of the lytic effect of complement and anti-microbial peptides [47]. In recent years, many studies have attempted to use phage depolymerase to treat capsular bacterial infections. Lin et al. constructed a mouse infection model by intraperitoneal injection of Klebsiella pneumoniae and injected the depolymerase K1-ORF34 30 min after infection, and in 30-day observation, all mice survived and did not show obvious symptoms of infection [48]. This may be related to the enhanced immunosensitive of mice to pneumococcus after depolymerase inhibited the capsular polysaccharide of bacteria. Sensitization to serum killing after removal of CPS has been previously reported for *Acinetobacter baumannii*, *Klebsiella pneumoniae*, and *Providencia stuartii*, with apparently similar mechanisms [49,50,51,52]. To verify the effect of depolymerase on CPS, we carried out a combined experiment of depolymerase and human serum. The experimental results agree with previously reported results [53,54]. Compared with UA168 not treated with depolymerase, UA168 treated with depolymerase showed a statistically significant decrease in the number of colonies when mixed with different volumes of serum. When the ratio of depolymerase to human serum was 3:1, the number of bacteria decreased the most, and there was a statistically significant difference (*p* < 0.05), whereas neither heat-treated serum nor depolymerase alone could reduce the bacterial load. It is also consistent with the previous findings of Abdelkader et al. [55], who confirmed that serum-mediated and neutrophil-mediated cell-killing efficiencies were significantly increased in Acinetobacter baumannii MK34, which lacked capsular polysaccharide. DpoMK34 sensitized Acinetobacter baumannii MK34 to serum killing in a serum concentration-dependent manner, with complete eradication at 50% serum concentration. This effect was eliminated upon thermal inactivation of serum complement, and this study highlights the role of CPS in shielding serum complement from entry into the host. It was hypothesized that when depolymerase was applied to CRKP strains, it could disrupt the bacterial capsular polysaccharide to varying degrees, therefore increasing the susceptibility of immunoreactive substances (e.g., complement) in the serum to bacteria and increasing the phagocytosis of macrophages and enhancing the bactericidal effect of neutrophils (PMN) in the serum through immune conditioning. The heat-treated serum was unable to reduce the bacterial load due to the inactivation of its complement, even though the capsular ride was destroyed by depolymerase, corroborating the fact that complement plays an important role in serum.

However, there are some limitations in the application of depolymerase. First, as depolymerase can only destroy the extracellular polysaccharide of biofilm or weaken the role of capsular polysaccharide, the effect of depolymerase depends on the host’s immunity. In immunocompromised hosts, the effect of depolymerase may be greatly reduced. In addition, due to the specificity of the depolymerase to the capsule of the host bacteria, one depolymerase can only recognize a certain type of extracellular polysaccharide produced by the same bacteria, but not all types or cannot even decompose the extracellular polysaccharide polymer produced by the same strain under different living conditions. Therefore, more exploration and analysis of the mechanism of depolymerase are needed [56].

## 5. Conclusions

The combination of Phage168-encoded polysaccharide depolymerase with polymyxin B or human serum effectively disrupted CRKP biofilms and reduced bacterial load, warranting further investigation as a treatment for CRKP infections.

## Data Availability

All data generated or analyzed during this study are included in this published article.
